# Analysis of the Flavonoidome Reveals the Different Health-Promoting Flavonoid Characteristics in Fruit

**DOI:** 10.3390/antiox12091665

**Published:** 2023-08-24

**Authors:** Chi Zhang, Yao Zhao, Han Tao, Linying Li, Yuqing He, Xueying Zhang, Ying Zhu, Gaojie Hong

**Affiliations:** State Key Laboratory for Managing Biotic and Chemical Treats to the Quality and Safety of Agro-Products, Key Laboratory of Biotechnology in Plant Protection of MOA of China and Zhejiang Province, Institute of Virology and Biotechnology, Zhejiang Academy of Agricultural Sciences, Hangzhou 310021, China; zhangchi2015@sibs.ac.cn (C.Z.); taohan@zju.edu.cn (H.T.); heyuqing@zaas.ac.cn (Y.H.); zhangxueying@zaas.ac.cn (X.Z.); yzhuzaas@zaas.ac.cn (Y.Z.)

**Keywords:** phytochemicals, fruit, metabolomics, antioxidant, health-promoting

## Abstract

Flavonoids are one of the important metabolites of plants, and many flavonoids have functions of antioxidant or antimicrobial, which can help plants resist environmental stress. On the other hand, flavonoids also have a health-promoting effect for humans, such as antioxidant and anti-aging, and some flavonoids can assist in disease treatment. Fruit is one of the main sources of plant food and flavonoids intake for humans. Understanding the flavonoidome of various fruits is helpful to choose fruit combinations according to different demands. In this study, we explored the composition and relative content of flavonoids in 22 fruits and analyzed some health-promoting flavonoids in fruits. In addition, we selected several fruits and measured their antioxidant capacity through experiments. Our study initially established a database of fruit flavonoidome, and can provide reference for nutrition research, fruit breeding and industrial development.

## 1. Introduction

Fruit is one of the most important plant food sources for humans. Compared to vegetables, most fruits can be eaten without heating, and thus they retain more of their nutrients. Fruit contains a variety of dietary phytochemicals which can be roughly divided into alkaloids, phenolics, nitrogen-containing compounds, organosulfur compounds, phytosterols, and carotenoids [1]. Flavonoids are secondary metabolites and belong to the phenolics, which have been widely studied to have benefits in human health.

Flavonoids have three rings (C6-C3-C6) as their basic skeleton in chemical structure. Based on the oxidation degree of the central heterocycle, flavonoids can be classified to flavonols, flavones, isoflavones, anthocyanins, flavanones, flavanols, and chalcones [2]. Flavonoids are well known for their antioxidant activity due to the ability to reduce free radical formation and scavenge free radicals [3]. For plants, flavonoids can protect them from biotic or abiotic threats. For example, sakuranetin is a type of flavonoid phytoalexin which can inhibit the growth of the fungal pathogen *Magnapothe oryzae* in rice [4]. A higher content of anthocyanin and flavonoid can improve the cold tolerance of mango [5]. For consumers, many flavonoids are beneficial to human health. Studies show that quercetin and catechin have an anti-inflammatory effect on macrophages [6]. Researchers have also found that some flavonoids have anti-tumor activity such as morin, quercetin and rutin [7,8]. In these studies, flavonoids can inhibit the proliferation and migration of tumor cells and influence the expression of some tumor-related genes. As flavonoids have many benefits for health, many studies have analyzed flavonoids in various fruits such as jujube and blackberry [9,10]. However, these studies usually focus on a specific fruit, and there are still limited summaries and comparative analyses between various fruits. To understand more about the health-promoting effects of flavonoids in food, scientists have also studied the bioaccessibility of various flavonoids. For example, in animal studies, anthocyanin from fruits can be absorbed from the stomach and small intestine and the bioavailability is estimated to be 0.26–1.8% [11]. In addition, the form of food also affects the bioaccessibility of flavonoids. A study showed that hesperidin was more bioaccessible from orange juice than from orange segments [12]. Since a large number of flavonoids exist in plants, focusing on a single flavonoid or flavonoid in a single species limits a comprehensive understanding of the distribution and variety of flavonoids in foods, especially in fruits. We summarized and analyzed the composition, classification and relative content of flavonoids in different fruits, and summarized them as the flavonoidome. Exploring the flavonoidome by metabolomics contributes to understanding the composition and content of various flavonoids in different fruits.

Metabolomics is a useful tool to investigate the various metabolites in organisms [13]. Similar studies have been conducted to uncover the difference of nutrients between various vegetables [14]. However, there are still few comparative studies focusing on flavonoids in various fruits. Here, we analyze the flavonoidome in 22 common fruits in the south of China. We also selected several fruits to determine their antioxidant capacity. These results help us better understand the differences in the types and amounts of flavonoids found in different fruits and provide a reference for a healthy diet and industrial utilization of natural products.

## 2. Materials and Methods

### 2.1. Plant Materials

To study the flavonoidome of the fruit, 22 fruits, including pear (*Pyrus communis* L.), red bayberry (*Myrica rubra Siebold et Zuccarini*), grape (*Vitis Vinifera* L.), strawberry (*Fragaria × ananassa Duch.*), blueberry (*Vaccinium corymbosum* L.), cherry (*Prunus avium* L.), apple (*Malus pumila Mill.*), durian (*Durio zibethinus Murr.*), mandarin (*Citrus reticulate Blanco*), loquat (*Eriobotrya japonica* (*Thunb.*) *Lindl.*), litchi (*Litchi chinensis Sonn.*), kiwifruit (*Actinidia chinensis Planch.*), watermelon (*Citrullus lanatus* (*Thunb.*) *Matsum. et Nakai*), Hami melon (*Cucumis melo* L.), pineapple (*Ananas comosus* (L.) *Merr.*), papaya (*Carica papaya* L.), banana (*Musa sapientum* L.), passion fruit (*Passiflora edulis Sims*), mango (*Mangifera indica* L.), mangosteen (*Garcinia mangostana* L.), grapefruit (*Citrus × aurantium Linnaeus*) and pitaya (*Hylocereus undatus* (*Haw.*) *Britt. et Rose*), were bought from a local market in Hangzhou, China. Samples were harvested and frozen in liquid nitrogen for the metabolome analysis.

### 2.2. Sample Extraction

Samples were vacuum freeze-dried by a lyophilizer (ScientZ-100F, Ningbo, China) and ground (30 Hz,1.5 min) to a powder using a grinding instrument (MM 400, Retsch, Haan, Germany). One hundred milligram of powder was weighed and dissolved in 1.2 mL of 70% methanol extract. The samples were vortexed for 30 s every 30 min six times and stored at 4 °C overnight. After centrifugation at 12,000 rpm for 10 min, the supernatant was filtered through a microporous membrane with a pore size of 0.22 μm. Then, samples were transferred to an injection bottle for UPLC-MS/MS analysis.

### 2.3. Ultra-Performance Liquid Chromatography (UPLC) Analysis

The metabolome of flavonoids was analyzed by ultra-performance liquid chromatography (UPLC) using a SHIMADZU Nexera X2 (Kyoto, Japan). The chromatographic column, Agilent SBC18 1.8 µm, 2.1 mm × 100 mm, was used at a flow rate of 0.35 mL/min. The mobile phase A was ultra-pure water (0.1% formic acid added) and phase B was acetonitrile (0.1% formic acid added). The elution gradient was: B phase at 5% at the beginning, increasing linearly to 95% within 9 min, then this was maintained at 95% for 1 min; then, B phase decreases to 5% in 1 min, and equals 5% for 3 min. The column temperature was 40 °C. The loading sample volume was 4 μL. The effluent was alternatively connected to an ESI-triple quadrupole-linear ion trap (QTRAP)-MS.

### 2.4. ESI-QTRAP-MS/MS

Linear ion hydrazine-flight time (LIT) and triple quadrupole (QQQ) scanning were obtained on the triple quadrupole linear ion TRAP mass spectrometer AB4500 Q TRAP (Framingham, MA, USA). The UPLC/MS/MS system was equipped with an ESI turbo ion spray interface. Both positive and negative ion modes can be controlled by Analyst 1.6.3 software (AB Sciex). The ESI source operating parameters are as follows: ion source, turbo spray; source temperature, 550 °C; ion spray voltage (IS), 5500 V (positive ion mode)/−4500 V (negative ion mode); ion source gas I (GSI), gas II (GSII), and curtain gas (CUR) were set to 50, 60, and 25.0 psi, respectively; and collision-induced ionization parameters were set to high. The instrument was tuned and calibrated with 10 and 100 μmol/L propylene glycol solution in the QQQ and LIT modes, respectively. The QQQ scan uses an MRM mode and has the collision gas (nitrogen) set to medium. Through further DP and CE optimization, the DP and CE of each MRM ion pair were completed. A specific set of MRM ion pairs was monitored at each period, based on the metabolites eluted during each period.

### 2.5. Data Analysis of the Widely Targeted Metabolome

Based on the MWDB (Metware database), the material was qualitatively determined according to the secondary spectral information. The isotopic signals, repeated signals of K^+^, Na^+^, NH_4_^+^, and the repeated signals of large-molecularweight fragments were removed during the analysis. Data were analyzed and visualized by Metware Cloud (https://cloud.metware.cn/), accessed on 11 August 2023.

### 2.6. Determination of Antioxidant System Activity

Hydroxyl radicals were produced by Fenton reactions of H_2_O_2_/Fe^2+^, and the scavenging rate of hydroxyl radicals was determined by measuring the change in absorbance at 510 nm.

The ammonium molybdate method was used, and the change in absorbance at 405 nm was measured to determine the CAT (catalase) activity. One unit of CAT activity was defined as the amount of decomposed 1 µmol H_2_O_2_ per second per g of sample.

The nitrotetrazolium blue chloride (NBT) method was used, and the change in absorbance at 560 nm was measured to determine the SOD (superoxide dismutase) activity. When the inhibition rate in the reaction system is 50%, the activity of SOD in the reaction system is defined as one unit of enzyme activity (U/mL).

The H_2_O_2_ method was used, and the change in absorbance at 470 nm was measured to determine the POD (peroxidase) activity. A change in an absorbance value of 0.01 per minute per g of sample in a 1 mL reaction system is defined as one unit of enzyme activity.

The commercial kits (Cominbio Co., Ltd., Suzhou, China) were used to determine these antioxidant system activities. A microplate reader (INFINITE 200 PRO, TECAN) was used to measure the absorbance of each reaction mixture.

### 2.7. Statistical Analysis

Using the GraphPad Prism 8 statistical software, the data were analyzed using a one-way analysis of variance (ANOVA) followed by the least significant difference test at a 95% confidence level. To indicate statistically significant differences, different letters above the columns are used. In the figures, the standard error is depicted. The experiments were repeated three times, and three samples replicates were taken with similar results. The principal component analysis (PCA) was also performed using R software (v. 3.4.1). The data were standardized and PCA analysis was performed using the R software’s own principal component analysis function “prcomp”.

## 3. Results

### 3.1. Detect the Flavonoidome of Fruits

**Figure 1:**
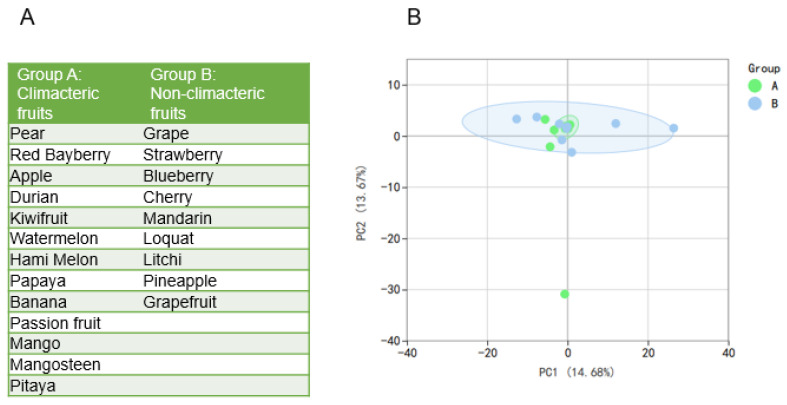
Principal component analysis (PCA) of fruit flavonoids. (**A**) Group of 22 fruits according to their ripening process. (**B**) PCA of the total flavonoids in 22 fruits.

To obtain a deeper understanding of the flavonoidome of different fruits, a widely targeted metabolome assay was performed on 22 fruits commonly sold in China. According to the ripening process, fruits can be divided into climacteric fruits and non-climacteric fruits, and different phytohormones regulate the ripening process in these two kinds of fruit [15]. As phytohormones can influence the synthesis of flavonoids, we want to explore whether the ripening process is related to the flavonoidome. These 22 fruits can be divided into climacteric fruit, including pear (*Pyrus communis* L.), red bayberry (*Myrica rubra Siebold et Zuccarini*), apple (*Malus pumila Mill.*), durian (*Durio zibethinus Murr.*), kiwifruit (*Actinidia chinensis Planch.*), watermelon (*Citrullus lanatus* (*Thunb.*) *Matsum. et Nakai*), Hami melon (*Cucumis melo* L.), papaya (*Carica papaya* L.), banana (*Musa sapientum* L.), passion fruit (*Passiflora edulis Sims*), mango (*Mangifera indica* L.), mangosteen (*Garcinia mangostana* L.) and pitaya (*Hylocereus undatus* (*Haw.*) *Britt. et Rose*); and non-climacteric fruit, including grape (*Vitis Vinifera* L.), strawberry (*Fragaria × ananassa Duch.*), blueberry (*Vaccinium corymbosum* L.), cherry (*Prunus avium* L.), mandarin (*Citrus reticulate Blanco*), loquat (*Eriobotrya japonica* (*Thunb.*) *Lindl.*), litchi (*Litchi chinensis Sonn.*), pineapple (*Ananas comosus* (L.) *Merr.*) and grapefruit (*Citrus × aurantium Linnaeus*) (Figure 1A). To explore whether the flavonoidome in fruit is correlated with the different ripening process, we performed a PCA analysis of these fruits. The result showed that component 1 (PC1) explained 14.68% and component 2 (PC2) explained 13.67% of the variability. These data show that most fruits have similar principal components of flavonoids, except pear (Figure 1B). The metabolic diversity of other fruits is non-significant since they clustered together in the PCA score plots. This result shows that there was no correlation between the components of the flavonoids and the ripening process (climacteric or non-climacteric).

**Figure 2:**
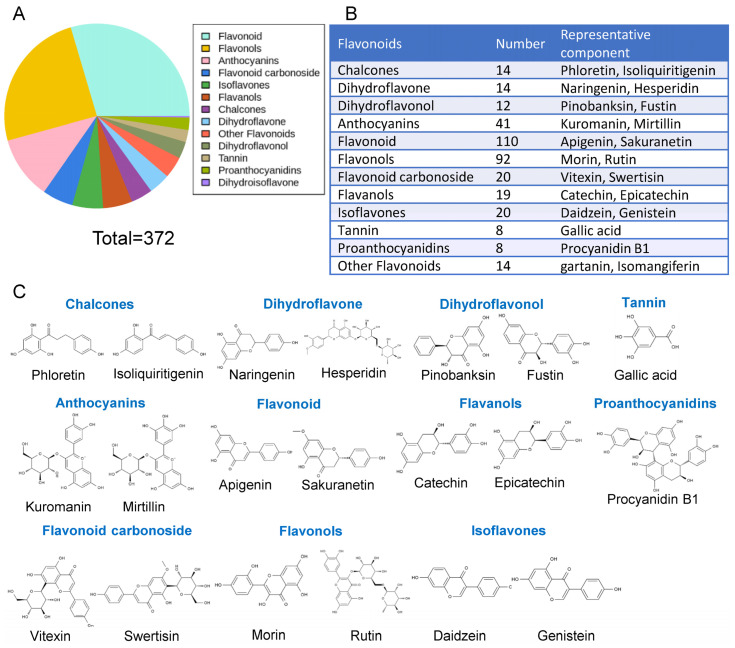
Classification and statistics of flavonoids in fruit. (**A**) Classification of the total detected flavonoids. (**B**) Statistics and examples of flavonoids. (**C**) Structural formula of flavonoids in (**B**).

In the metabolome data, a total of 372 flavonoids were detected (Figure 2A). These flavonoids were classified as chalcones, flavanones, dihydroflavonol, anthocyanins, flavones, flavonols, flavonoid carbonoside, flavanols and isoflavones. Though tannin and proanthocyanidins do not belong to flavonoids, they were also detected for technical reasons. Among these flavonoids, flavone is the most abundant, with 110 species, and the second most abundant were flavonols, with 92 species. There were 14 flavonoids that did not fall into any of these categories, and therefore they were classified as other flavonoids (Figure 2B). Some of these flavonoids, such as apigenin and sakuranetin, have been extensively studied and found to be beneficial to human health, and Figure 2C shows their structure. As these flavonoids may have a potential effect on human health, it is important to understand the specific types and amounts of flavonoids in different fruits.

### 3.2. Comparative Analysis of Flavonoids in 22 Fruits

**Figure 3:**
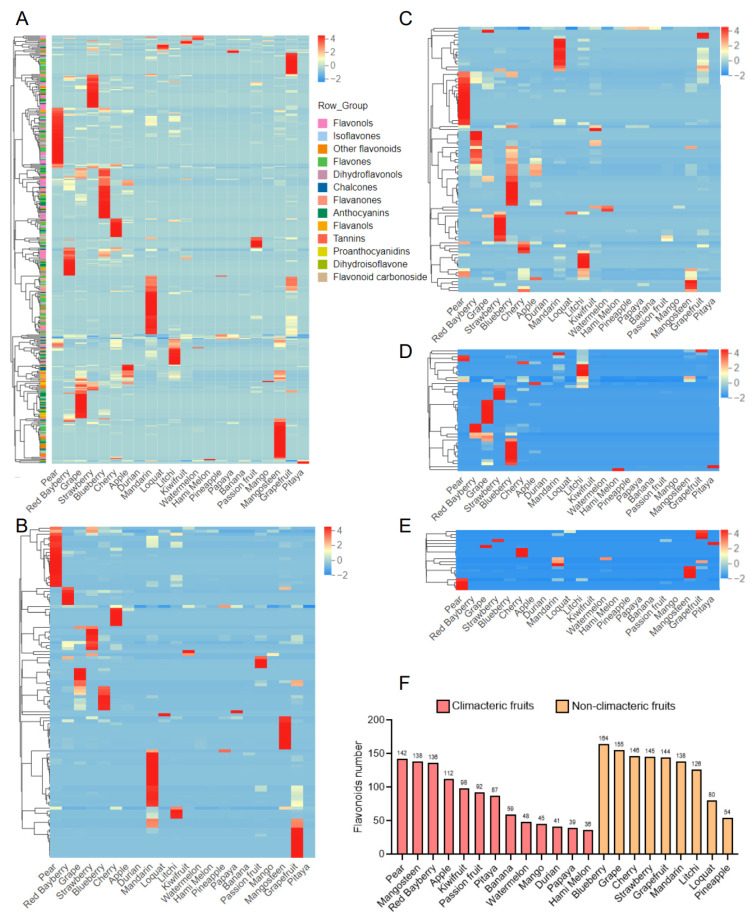
Distribution of flavonoid species in each fruit. (**A**) Cluster heatmap of the total flavonoids. (**B**) Cluster heatmap of the total flavonoids. (**C**) Cluster heatmap of the total flavonols. (**D**) Cluster heatmap of the total anthocyanins. (**E**) Cluster heatmap of the total isoflavones. (**F**) The total number of flavonoids in each fruit.

To illustrate the distribution of flavonoids in 22 fruits, hierarchical cluster analysis was used to visualize the relative abundance of flavonoids. In the cluster heatmap of the flavonoidome, pear, blueberry, mandarin, mangosteen, grape, strawberry, grapefruit and litchi exhibited a relative higher abundance of flavonoids (Figure 3A). As flavones, flavonols, anthocyanins and isoflavones are the four most abundant groups in the total flavonoids, we also carried out heatmap analysis of the four groups separately (Figure 3B–D). The heatmap of the flavones showed that pear, mandarin, grapefruit and mangosteen had a higher abundance (Figure 3B). In the cluster heatmap of flavonols, pear, blueberry, mandarin, red bayberry and strawberry were shown to contain a higher amount (Figure 3C). Anthocyanins are one of the most important groups of substances that give color to plants [16]. In our data, two dark fruit, blueberry and grape, had a higher abundance of anthocyanins (Figure 3D). As for the isoflavones, pear, mangosteen, cherry and grapefruit showed the highest abundance in the heatmap (Figure 3E).

To better understand the difference of flavonoids in the 22 fruits, we counted the total number and distribution of flavonoids in various fruits. In terms of flavonoid diversity, blueberry had the widest variety with 164 flavonoids, while grape (155), cherry (146), strawberry (145), and grapefruit (144) followed (Figure 3F). Hami melon, papaya, durian, mango and watermelon had fewer flavonoids, with 36, 39, 41, 45 and 48, respectively (Figure 3F). Intriguingly, the non-climacteric fruits contained a greater variety of flavonoids on average.

We also counted the proportion of various flavonoids in the 22 fruits. Like the data in Figure 2A, flavones and flavonols were the two most common kinds of flavonoids in all the 22 fruits. Though the number of flavones accounted is more in the total flavonoids, the number of flavonols was higher in many fruits, such as red bayberry, grape and strawberry (Figure S1). Additionally, the number of anthocyanins was relatively low in watermelon, passion fruit, mango and grapefruit (Figure S1). These results help us understand the differences in the composition of flavonoids in different fruits and can be used as a reference for raw materials of flavonoid extraction.

### 3.3. Health-Promoting Flavonoids in Fruit

The composition and proportion of flavonoids varied from fruit to fruit. In addition, we found that some fruits contained flavonoids not present in other fruits (Table 1). In our data, isoliquiritigenin was only present in passion fruit, belonging to chalcones, and this study showed that it has the ability to carry out antigrowth and -proliferation in various cancer cells [17]. Mangosteen contained many unique flavonoids such as garcinone B and kushenol E, and research has found that they have potential activity in anti-microbial and cancer therapy, respectively [18,19]. Though there is not too much research about these flavonoids in mangosteen, they remain a potential biological resource for food and medical research. Apigenin, wogonin and chrysosplenol D belong to flavone, and they are specifically present in cherry, with studies showing they all have antitumor activity [20,21,22]. Jaceosidin is a unique flavone in red bayberry. Studies in mice found that it can inhibit myocardial oxidative damage and the inflammatory response [23]. Astragalin is a flavonol found in pear, which functions in ulcerative colitis therapy [24].

**Figure 4:**
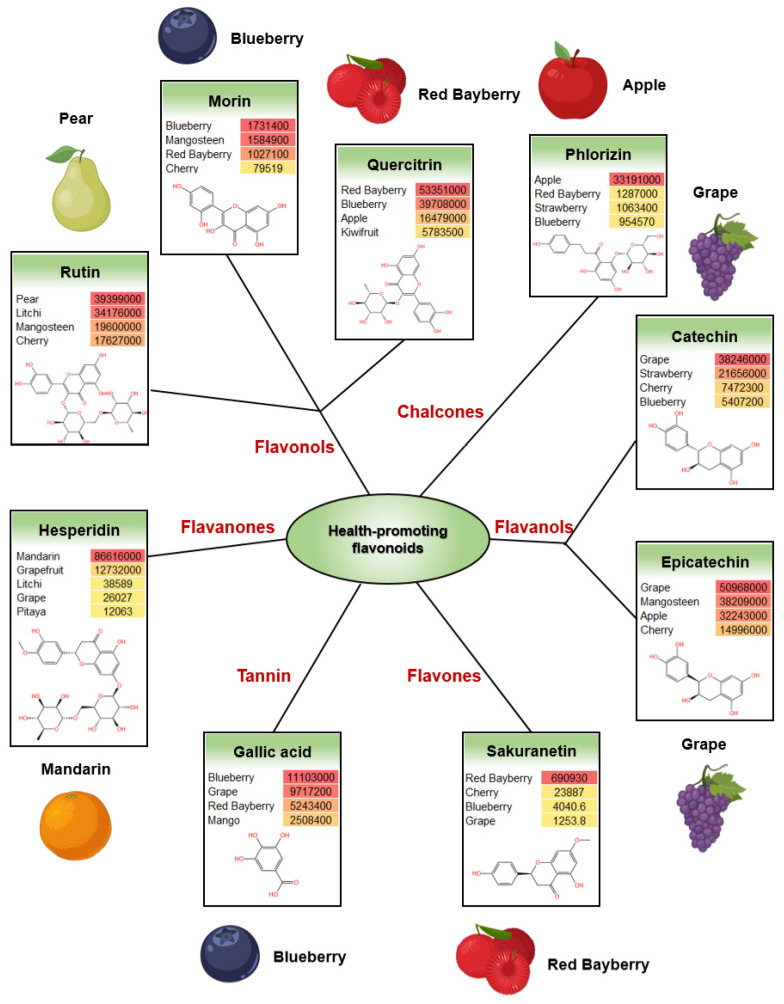
Some common health-promoting flavonoids in fruit.

In addition to the unique flavonoids found in certain fruits, there are also some health-promoting flavonoids that are common in many fruits. Here, we list some of the health-promoting flavonoids and the fruits that contain more of them. Flavonols are one of the most common kinds of flavonoids. Some flavonols with health-promoting functions have been well studied, such as rutin, morin and quercitrin. Rutin is widely found in many fruits, and studies have shown that it has anti-diabetic and anti-Alzheimer’s effects [25,26]. In our data, 19 fruit contain rutin, and the highest levels are found in pear and litchi (Figure 4). Morin was found in eight fruit, and the amount in blueberry and mangosteen was the most (Figure 4). These flavonols have multiple functions including antioxidant, anti-diabetic, anti-inflammatory, antitumor, antihypertensive and antibacterial effects [27]. Quercitrin is another flavonol with a wide spectrum of bioactivities including antioxidant, anti-inflammation, anti-microbial and immunomodulation [28]. It was found in 14 kinds of fruit, including red bayberry and blueberry (Figure 4). Phlorizin belongs to chalcones, it was most abundant in apple (Figure 4) and has antioxidant and anti-inflammatory activities [29]. Red bayberry contained the most sakuranetin in the 22 fruits (Figure 4), and this flavonoid is reported to have antioxidant, anti-microbial and anti-inflammatory effects [30]. Catechin and epicatechin are both flavanols with antioxidant and anti-inflammation functions [31], and are well known in tea. In our data, they were abundant in grape (Figure 4). Gallic acid is a hydroxybenzoic acid. It is found in both a free state or as a constituent of hydrolysable tannins, and it is a widely used food additive and industrial auxiliary material. It appeared in 13 of the fruits and has antioxidant, anti-cancer and anti-microbial abilities (Figure 4) [32]. The flavanone hesperidin was mainly found in citrus fruits such as mandarin and grapefruit (Figure 4). It has health-promoting effects like anti-inflammation, antioxidant and lipid-lowering effects [33].

### 3.4. Antioxidant System Activities in Different Fruit

**Figure 5:**
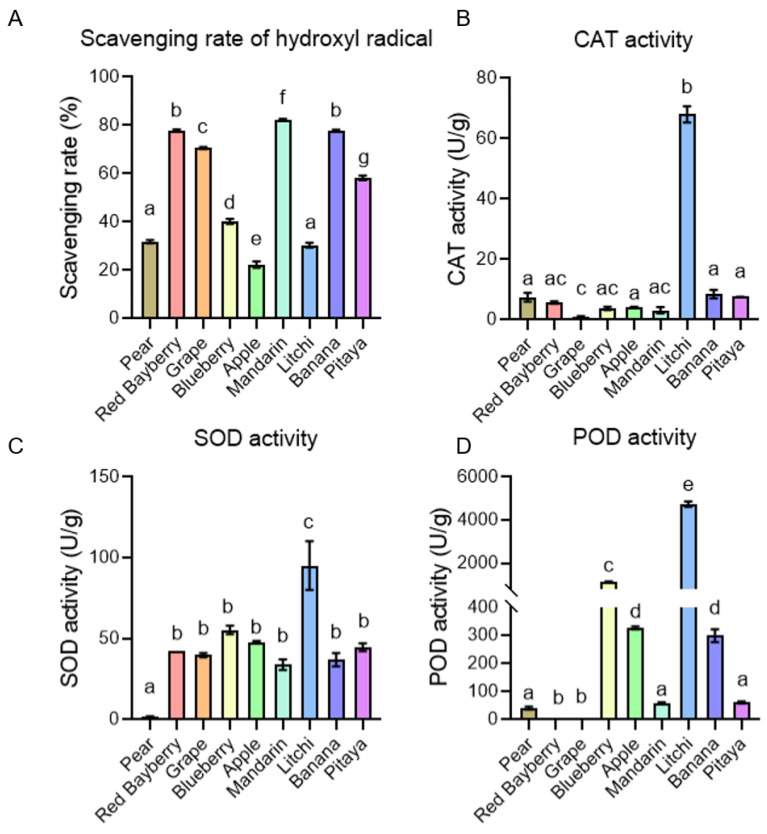
The activity of the antioxidant enzymes in the different fruits. (**A**) Scavenging rate of the hydroxyl radical in the fruits. (**B**) CAT activity in the fruits. (**C**) SOD activity in the fruits. (**D**) POD activity in the fruits. Error bars represent the SE. Letters indicate significant difference assessed by one-way ANOVA, *p* < 0.05.

Many of the flavonoids mentioned above have antioxidant properties. Due to the lower redox potentials, flavonoids can reduce highly oxidizing free radicals. In addition, flavonoids can form complexes with free radicals involved in oxidative processes and stabilize them [3]. To investigate the antioxidant ability of the fruits, we chose nine fruits that were rich in flavonoids, including pear, red bayberry, grape, blueberry, apple, mandarin litchi, banana and pitaya, then detected the scavenging rate of hydroxyl radicals, and the antioxidative enzyme activity of catalase (CAT), superoxide dismutase (SOD) and peroxidase (POD). The hydroxyl radical is the most powerful but short-lived reactive oxygen species in plants. Though it can also function as a signal molecule, its destructive effect is unable to be ignored [34]. So, the redundant hydroxyl radical should be effectively scavenged. We detected the hydroxyl radical removal ability of these nine fruits, and the results showed that the extracts of mandarin, red bayberry and banana exhibited the highest scavenging rate (Figure 5A). SOD and CAT are two enzymes that degrade superoxide anion (O_2_^•−^) and hydrogen peroxide (H_2_O_2_), respectively. The superoxide anion can be dismutated to hydrogen peroxide and O_2_ by SOD, and hydrogen peroxide can be scavenged by CAT [35]. In these nine fruits, the extract of litchi showed the highest CAT and SOD activity (Figure 5B,C). Peroxidase is a superfamily of enzymes which can reduce peroxide through substrate oxidation, and thus protect plant from oxidative stress [36]. Like the results of CAT and SOD activity, litchi had the highest POD activity among these nine fruits (Figure 5D).

## 4. Discussion

Although fruit is not a substitute for medicine, the range of contained flavonoids still has the potential to promote human health. Furthermore, fruit can also be used as a biological source of industrially extracted compounds. By metabolome analysis of fruits, it is possible to understand which metabolites are enriched in which fruits. This will provide a reference for extracting and purifying compounds that are difficult to synthesize artificially. In addition, metabolome analysis can also identify potential allergens or compounds that are harmful to humans. By modifying the metabolic pathway genes in fruit, safer and tastier fruits could be created. In this study, we divided the fruits into climacteric and non-climacteric, and tried to explore whether the ripening process can affect the components of the flavonoids. However, our PCA analysis showed that both ripening processes showed no significant difference in the components of the flavonoids. The ripening mode of the fruit is mainly regulated by plant hormones, while flavonoids help plants adapt to the environment and regulate their physiological state, and this may have limited influence on the ripening process.

In the antioxidant ability experiment, litchi showed the highest activity of CAT, SOD and POD. We listed the top 40 flavonoids with the highest content in litchi, which belong to anthocyanins, flavonols, flavones, isoflavones, proanthocyanidins, flavonoid carbonoside and chalcones (Table S1). Although there was no direct evidence that they affect the activity of CAT, SOD and POD enzymes in litchi, their biological functions and potential applications are still worth exploring. A study has shown that in addition to flavonoids, litchi is also rich in phenolic acids, which may also be one of the reasons for its high antioxidant activity [37].

Flavonoids belong to phenolics, and they are not the only metabolites with antioxidant abilities. Other phenolics, carotenoids and polyphenolics are all reported to have a significant role in plant antioxidant responses [38,39]. Therefore, the type and content of fruit flavonoids play an important role but it cannot be directly used to measure their antioxidant capacity. Besides antioxidants, flavonoids also have many beneficial effects in regulating physiological indicators and assisting in disease treatment. The discovery of the fruit flavonoidome can provide a reference for future research in these aspects. For plants, the synthesis of flavonoids ensures that they grow better and protect themselves from invasion. Thus, scientists can modify the flavonoids synthesis pathway to breed more disease-resistant and storage-resistant fruits.

Though flavonoids have lots of benefit for human health, a relative low bioaccessibility limits the body’s absorption of flavonoids from food. Therefore, the health-promoting effects of flavonoids are not entirely utilized through digestion and absorption. The intestine is the main organ of the human body that absorbs nutrition from food, and various gut microbes in the intestine directly or indirectly participate in the digestion and absorption of nutrients, playing an important role in health. Changes in diet and abuse of antibiotics can alter the microbial composition and lead to different health outcomes. The flavonoids in food are hydrolyzed and absorbed in the gut and are bound to glucuronate/sulfate forms by phase II enzymes in epithelial cells and the liver. The gut microbes play an important role in the metabolism of dietary flavonoids [40]. A study has shown that the flavonoid quercetin can promote populations of *Bifidobacterium*, *Bacteroides*, *Clostridia*, and *Lactobacillus* and significantly suppressed *Enterococcus* and *Fusobacterium*, thus promoting gut homeostasis [41]. Catechins are one of the main antioxidant agents, and they are widely distributed in many foods, especially green tea. The metabolism of catechins requires gut microbes, which can biotransform catechins into phenylvalerolactones and phenylvaleric acids [42]. On the other hand, catechins can inhibit the growth of *Helicobacter pylori*, *Staphylococcus aureus*, *Escherichia coli* O157:H7, *Salmonella typhimurium* DT104 and *Pseudomonas aeruginosa* [43]. These studies suggest that gut microbes can promote the metabolism of flavonoids, and flavonoids can also regulate gut microbiota to influence health.

## 5. Conclusions

In this study, we analyzed the flavonoidome in 22 fruits. There were total 372 flavonoids detected and divided into 12 groups (chalcones, flavanones, dihydroflavonol, anthocyanins, flavones, flavonols, flavonoid carbonoside, flavanols, isoflavones, tannin, proanthocyanidins and other flavonoids). Flavones and flavonols were the two most abundant flavonoids in all these 22 fruits; among them, pear and mandarin contained the most abundant flavones, and pear and blueberry contained the most abundant flavonols. Furthermore, we analyzed the content and benefits of some representative flavonols, chalcones, flavones, flavanols, tannin and flavanones in the fruits to cite their health-promoting effects. Then, we detected the antioxidant ability of some fruits and found that litchi had the highest antioxidant activity. Thus, our flavonoidome database can provide a reference for fruit genetic breeding and transformation, nutrition research and industrial development.

## Figures and Tables

**Table 1:** Specific flavonoids in some fruit.

Fruits	ID	Q1 (Da)	Q3 (Da)	Formula	Compounds	Class
Passion fruit	pme3217	257.08	137	C_15_H_12_O_4_	Isoliquiritigenin	Chalcones
	Lmmp007480	257.08	137.02	C_15_H_12_O_4_	2,4,4′-trihydroxychalcone	Flavones
	mws1292	565.16	409.09	C_26_H_28_O_14_	Isoschaftoside	Flavonoid carbonoside
Mangosteen	pmp000358	355.15	299.06	C_21_H_22_O_5_	Licoagrochalcone D	Chalcones
	Lmsp010391	329.1	287.06	C_18_H_16_O_6_	Salvigenin	Flavones
	pmp000638	355.12	299.06	C_20_H_18_O_6_	8-prenylkaempferol	Flavones
	Hsmp10763	381.17	269.05	C_23_H_24_O_5_	Mangostinone	Other Flavonoids
	Hsmp10672	395.15	339.09	C_23_H_22_O_6_	Garcinone B	Other Flavonoids
	Hsmp10144	409.17	353.1	C_24_H_24_O_6_	Mangostanin	Other Flavonoids
	pmp000385	423.22	367.12	C_26_H_30_O_5_	Kanzonol J	Other Flavonoids
	pmp000647	425.2	369.13	C_25_H_28_O_6_	Kushenol E	Other Flavonoids
	Hsmp09614	427.18	371.11	C_24_H_26_O_7_	Mangostanol	Other Flavonoids
	Hsmp08787	429.19	355.12	C_24_H_28_O_7_	Garcinone D	Other Flavonoids
	pmp000368	383.15	327.16	C_22_H_22_O_6_	Licoricone	Isoflavones
	Lmdp003994	461.14	299.09	C_23_H_24_O_10_	Wistin	Isoflavones
Cherry	pmp000383	419.13	257.08	C_21_H_22_O_9_	Liquiritin	Flavanones
	pmp000571	271.06	153.01	C_15_H_10_O_5_	Apigenin	Flavones
	mws4160	285.08	270.07	C_16_H_12_O_5_	Wogonin	Flavones
	Lmjp005224	361.09	328.06	C_18_H_16_O_8_	Jaceidin	Flavones
	pmp000788	361.09	346.06	C_18_H_16_O_8_	Chrysosplenol D	Flavonols
	pme3250	285.08	270	C_16_H_12_O_5_	Biochanin A	Isoflavones
	mws0908	283.06	268	C_16_H_12_O_5_	Glycitein	Isoflavones
Red Bayberry	pmp000234	527.15	331.08	C_27_H_26_O_11_	Salcolin A	Flavones
	pmp000004	331.08	316.06	C_17_H_14_O_7_	Jaceosidin	Flavones
Pear	mws2209	449.11	287.06	C_21_H_20_O_11_	Astragalin	Flavonols

## Data Availability

Data are contained within the article.

## Supplementary Materials

The following supporting information can be downloaded at: https://www.mdpi.com/article/10.3390/antiox12091665/s1, Figure S1: Classification of flavonoids in each fruit; Table S1: Top 40 flavonoids with the highest content in litchi.
