# Supplementary figures and images for: Analysis of the Flavonoidome Reveals the Different Health-Promoting Flavonoid Characteristics in Fruit
Source: Antioxidants (Basel). 2023 Aug 24;12(9):1665. doi: 10.3390/antiox12091665 (PMC10525919; doi:10.3390/antiox12091665)

## Slide 1
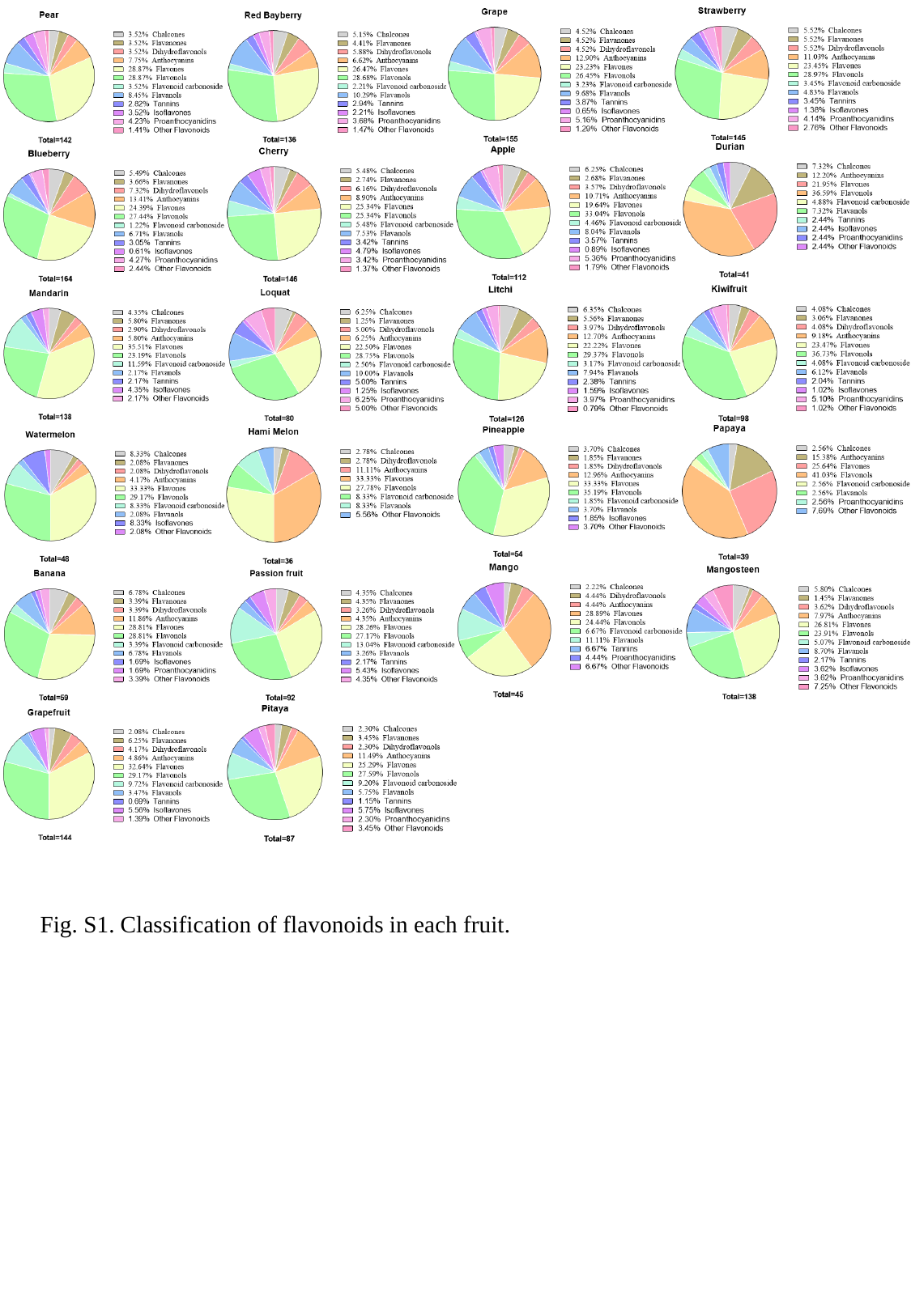

Fig. S1. Classification of flavonoids in each fruit.

Supplement: Supplementary file 1 [file antioxidants-12-01665-s001.zip › Supplementary Figure.pptx]
